# Effective Temperature and Universal Conductivity Scaling in Organic Semiconductors

**DOI:** 10.1038/srep16870

**Published:** 2015-11-19

**Authors:** Hassan Abdalla, Kevin van de Ruit, Martijn Kemerink

**Affiliations:** 1 Complex Materials and Devices, Department of Physics, Chemistry and Biology, Linköping University, 58183 Linköping, Sweden; 2 Eindhoven University of Technology, Department of Applied Physics, P.O. Box 513, NL-5600 MB Eindhoven, the Netherlands

## Abstract

We investigate the scalability of the temperature- and electric field-dependence of the conductivity of disordered organic semiconductors to ‘universal’ curves by two different but commonly employed methods; by so-called universal scaling and by using the effective temperature concept. Experimentally both scaling methods were found to be equally applicable to the out-of-plane charge transport in PEDOT:PSS thin films of various compositions. Both methods are shown to be equivalent in terms of functional dependence and to have identical limiting behavior. The experimentally observed scaling behavior can be reproduced by a numerical nearest-neighbor hopping model, accounting for the Coulomb interaction, the high charge carrier concentration and the energetic disorder. The underlying physics can be captured in a simple empirical model, describing the effective temperature of the charge carrier distribution as the outcome of a heat balance between Joule heating and (effective) temperature-dependent energy loss to the lattice.

Since the discovery of conductive polymers, tremendous progress has been made in rationalizing their electrical properties. At low charge carrier concentrations, typically stretched exponential behavior of the current *j* on temperature *T*, 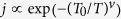, is found and rationalized in terms of hopping in a disorder-broadened density of states (DOS), with the system dimensionality and DOS shape determining the stretching exponent *v*^1^,^2^. The Ohmic conductivity of some conducting polymer systems with higher conductivity tends to show a power law temperature dependence, *σ* ∝ *T*^*α*^, typically considered to be a consequence of the increasingly metallic properties of the system, causing an insulator-to-metal transition^3^,^4^,^5^.

For higher carrier concentrations and associated higher conductivities the situation is not so clear. Many recent studies on organic electronic systems, which do not only measure the Ohmic conductivity but also the non-Ohmic conductivity obtained at increased electric fields, reveal a curious pattern^6^,^7^,^8^,^9^,^10^,^11^,^12^. They show that the Ohmic conductivity has a power law temperature dependence, but most importantly, that rescaling the current and voltage using the power law dependence on temperature collapses the data on a universal curve of *j*/*T*^1+*α*^ vs. *V*/*T* that can be captured by


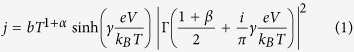


with *e* the elementary charge, *k*_*B*_ the Boltzmann constant, *b* a scaling parameter, *γ* a parameter such that the crossover between the Ohmic and non-Ohmic regime takes place at *eV*/*k*_*B*_*T* ≈ 2/*γ*, and Γ the complex Gamma function. (NB: the exponent in Eq. (1) is missing in ref. 6) The parameters *α* and *β* reflect the power law temperature and field dependence in the ohmic and non-ohmic regimes, respectively, and are often related as *β* = *α* + 1. The scaled curve consists of a crossover between an Ohmic voltage dependence, *j* ∝ *V*, and a steeper power law dependence, *j* ∝ *V*^*β*^. The property of obeying this scaling behavior is commonly referred to as universal scaling (US); accordingly in this work we shall refer to US as the property that the scaled *j*(*V*, *T*) curves exhibit these limiting power law behaviors, not necessarily obeying the exact form Eq. (1).

There are at least five different theoretical frameworks that either exactly explain the functional shape (1), or give rise to functionally similar behavior. In most experimental investigations observations of US behavior are explained as stemming from Luttinger liquid behavior, which is related to interacting electrons confined to one dimension^13^. Because of the strong similarity in the resulting charge transport behavior, a strong case can also be made for a description based on a chain of quantum dots with Coulomb blockade behavior^7^,^14^,^15^. Alternatively, Rodin and Fogler^16^ have shown that the power law behavior of conductance might also be well explained in terms of quasi-1D variable range hopping (VRH). Asadi *et al.* showed universal scaling for a wide range of semiconducting polymers and interpreted the results in terms of a model that accounts for the zero-point energy of the charges and that holds for low disorder^17^. Finally, Li *et al.* argued that in the high-density regime 3D VRH can give rise to behavior resembling Eq. (1) 18. No consensus exists regarding which of these prevails. Moreover, as these interpretations are based on fundamentally different and mutually exclusive assumptions, it is safe to conclude that the high-density conductivity of disordered organic semiconductors still poses a major question.

A seemingly unrelated concept that applies to amorphous semiconductors at high electric fields is the so-called effective temperature *T*_*eff*_. The effective temperature framework was first described some 20 years ago by Marianer and Shklovskii (MS) and confirmed by Baranovskii *et al.*, for inorganic amorphous systems with an exponential density of states (DOS) in the Boltzmann limit^19^,^20^. It has since been shown to be also valid for disordered organic systems with a Gaussian DOS and large charge densities^21^,^22^,^23^. In their original work, MS found that a strong enough electric field has a similar effect on the charge carrier population and transport as an increased lattice temperature. The underlying physical picture is that the carrier can increase its energy by an amount Δ*E* = *qFa* by hopping a distance *a* along the field *F*. In other words, the carrier becomes “hot”. They argue that the temperature in the Miller Abrahams expression for hopping conduction can then be replaced by an effective temperature, which is dependent on the applied field and the lattice temperature. In their numerical variable range hopping study of the effective temperature as a function of lattice temperature and field, they find that the variation of *T*_*eff*_/*T* with *qFa*/*k*_*B*_*T* for different temperatures collapses to one line with a functional shape following the expression


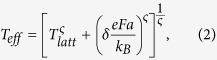


with *δ* = 0.67 and *ς* = 2. Marianer and Shklovskii write that they ‘unfortunately do not have a satisfactory physical interpretation’ of Eq. (2). Even though the concept of an effective temperature is by now theoretically well-established ‘as a rough but useful approximation’^23^,^24^,^25^, the functional shape of Eq. (2) remains unexplained, both for inorganic and organic systems. Remarkably, when the conductivity obtained from these numerical simulations is plotted as a function of *T*_*eff*_ another universal curve is obtained, with all data points following a power law in *T*_*eff*_, 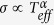. Also here no physical explanation is given. Experimentally, the validity of Eq. (2) has subsequently been confirmed by dark conductivity measurements of inorganic a-Si:H^26^,^27^,^28^; for organic semiconductors the concept received no experimental attention.

Despite evident similarities between the US and MS concepts, we are not aware of any attempts to unify both frameworks or to address a possible common physical origin. Here we show that the governing relations of universal scaling and effective temperature lead to fully equivalent functional shapes in temperature- and field-dependence of the conductivity and therefore equally well describe an extensive data set obtained on a practically relevant model system, the highly doped organic semiconductor PEDOT:PSS. Using kinetic Monte Carlo (MC) simulations we show that, in the experimentally relevant limit of large Gaussian disorder and high carrier concentrations, the US and MS scaling phenomena have their physical origin in the heating of the charge carrier distribution. The MC simulations are able to qualitatively reproduce all experimentally observed phenomena and relations. Furthermore, the time-dependent nature of the MC method allows us to investigate the energy relaxation of the charge carriers over time. Based on this knowledge we show how Marianer-Shklovskii-type behavior Eq. (2) and universal scaling can be obtained using a simple heat balance, based on Joule heating in combination with an algebraic energy dependence of the relaxation of charge carriers. The resulting empirical model consistently describes the numerical and experimental results.

## Results

As an experimental basis for our work, we investigated the out-of-plane conductivity of PEDOT:PSS thin films as a function of electric field, temperature, layer thickness and PEDOT:PSS weight ratio. The samples for the out-of-plane conductivity measurements were fabricated as described previously^29^. A summary is given in the Methods section at the end.

**Figure 1:**
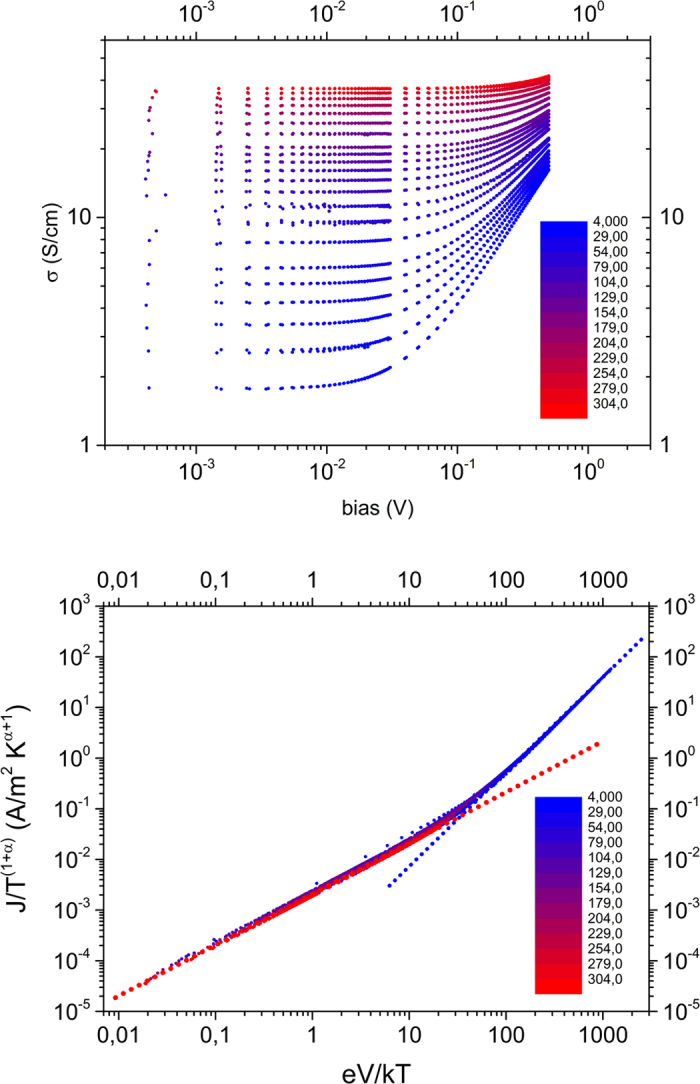
Universal scaling of the conductivity of PEDOT:PSS. (**a**) Conductivity vs. applied bias for a 1:2.5 (w/w) PEDOT:PSS thin film (thickness 50 nm, sample 1), measured at different temperatures indicated by the color scale. (**b**) Same data as in (**a**), rescaled according to the universal scaling procedure discussed in the text. The red and blue dotted lines indicate power laws with slope 1 and *β*, respectively; *α* = 1.0.

In Fig. 1a the conductivity of a 1:2.5 PEDOT:PSS thin film is plotted as a function of the applied bias voltage for temperatures ranging from 4 K to around 310 K. One can clearly see how the slope of the non-Ohmic behavior increases with decreasing temperature. The transition point at which the conductivity becomes field-dependent, i.e. non-ohmic, shifts to lower fields at lower temperatures. The low-field-conductivity plotted as a function of the temperature on a double-log scale shows a good linear behavior (see SI), from which we obtain the exponent α = 1.0 in the power law fit to the Ohmic data. If the current of the whole set of measurements is scaled to *j*/*T*^1+*α*^ and plotted as a function of the dimensionless ratio of field energy and thermal energy, *eV*/*k*_*B*_*T*, all points collapse to one line and a universal curve is obtained in accordance with Eq. (1), as shown in Fig. 1b. Said γ divides the universal curve into an Ohmic regime where *j* ∝ *T*^*α*^ and a temperature-independent non-ohmic regime where *j* ∝ *V*^*β*^. The exponents obey the relation *β* = *α* + 1 as described previously. Other samples investigated in the course of this work show an equally satisfying universal scaling behavior; the respective plots can be found in the SI.

**Figure 2:**
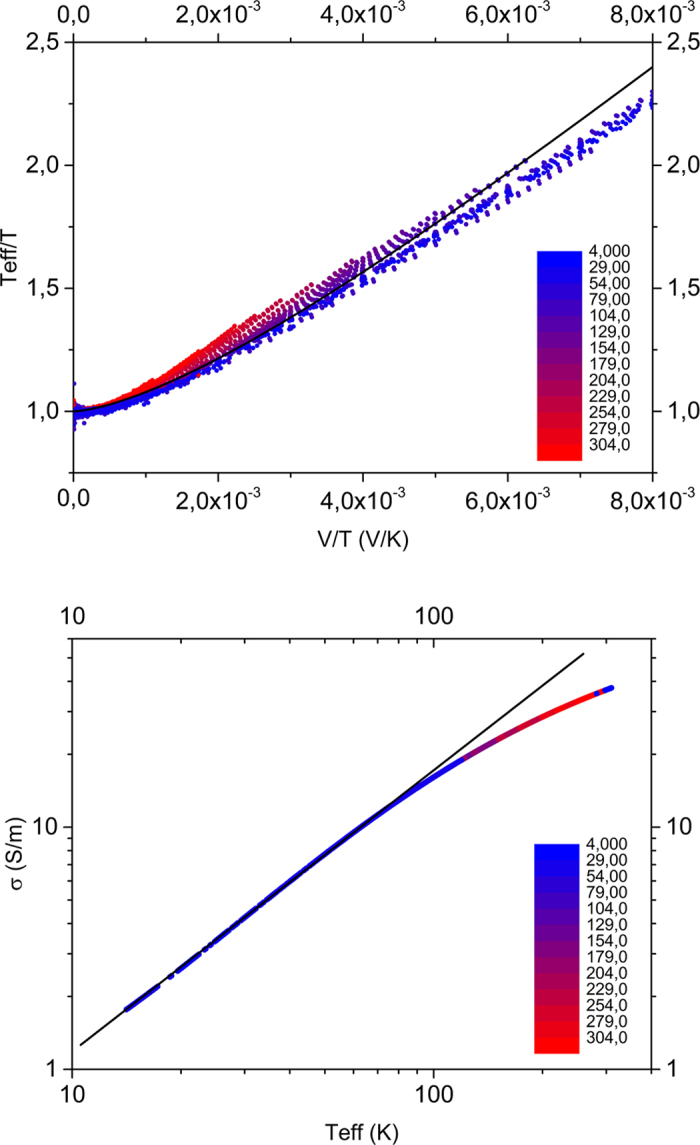
Effective temperature and correspondingly scaled conductivity of PEDOT:PSS. (**a**) Open circles show the collapse of the effective temperature for all data points of sample 1 (c.f. Fig. 1) when plotted against bias voltage and scaled with lattice temperature. The solid line is a fit of Eq. 2 to the experimental data with *δ* = 0.67, *ς* = 1.5 and *a* = 1.8 *nm*. (**b**) Dependence of the measured conductivity on the effective temperature. The dashed line indicates 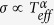 with *α* = 1.2.

Using the same set of data we investigated the temperature- and field-dependence in the effective temperature framework. As suggested by Marianer and Shklovskii^19^, *T*_*eff*_ is experimentally obtained in the following manner. The conductivity *σ*(*F*, *T*) at each field and temperature combination is compared to the temperature dependence of the Ohmic conductivity *σ*(0, *T*) to obtain the effective temperature from the condition *σ*(0, *T*_*eff*_) = *σ*(*F*, *T*). The practical implementation of solving this equality uses a numerical interpolation of the measured data points in the Ohmic regime, i.e. 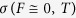. The resulting *T*_*eff*_ (*F*, *T*), seen in Fig. 2a, is a collapse of all data points to a single line following the functional shape of Eq. (2), which is plotted as a solid line with *ς* = 1.5 in accordance with ref. 21. Figure 2b shows the corresponding dependence of the measured conductivity on the effective temperature. In this case, the collapse of all points to a single line is an obvious consequence of the procedure followed to determine *T*_*eff*_. However, we find that the conductivity scales with a power law in *T*_*eff*_, 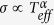, as indicated by the solid line, with minor deviations at high temperatures. We have no explanation for this behavior, but note that it is strikingly similar to the dependence of the current on the lattice temperature observed in the Ohmic part of the universal scaling curve in Fig. 1b.

**Figure 3:**
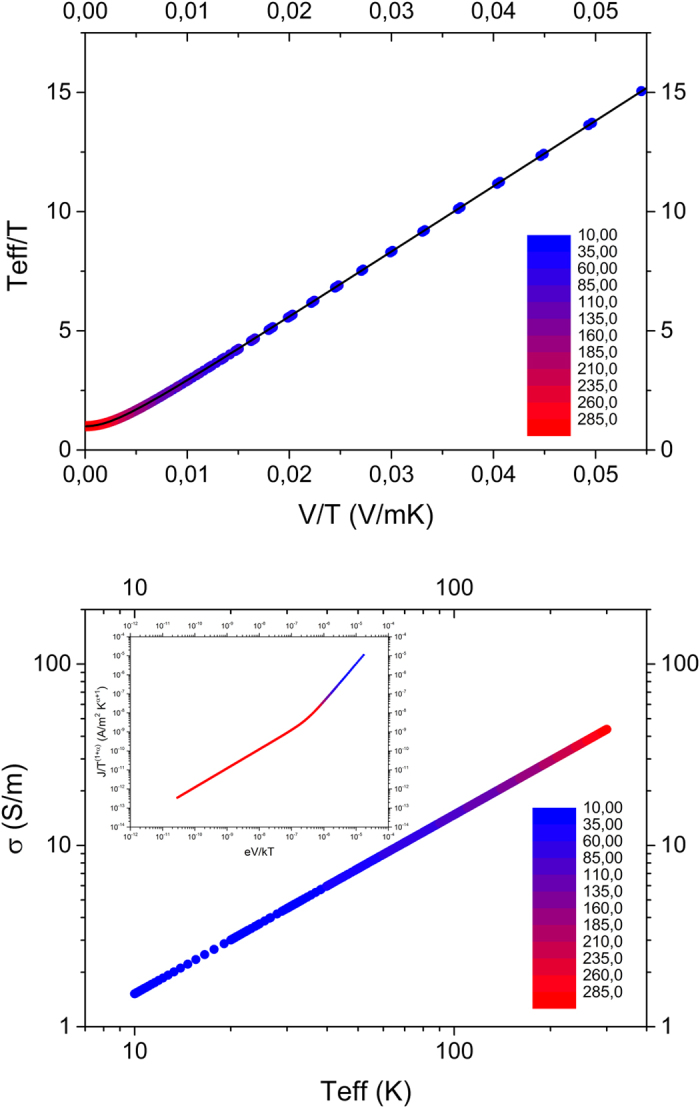
Effective temperature analysis of universal scaling behavior (Eq. 1). (**a**) Effective temperature vs. bias over temperature. Open circles are synthetic data generated from Eq. (1), the full line is a plot of the Marianer-Shklovskii expression Eq. (2), both with parameters corresponding to sample 1. (**b**) Conductivity vs. effective temperature for the same set of data. The inset shows the universal scaling behavior of the synthetic data from Eq. (1).

Summarizing our results so far, we have established that our experiments can be consistently analyzed in the US and MS frameworks, suggesting that these two frameworks may, in the present case, actually be equivalent. As a next step, we create synthetic IV-data from Eq. (1) with parameters obtained from fitting this equation to the universal scaling curve for sample 1 (Fig. 1b). We obtain and plot *T*_*eff*_ in the same manner as we did for the experiments, cf. Fig. 2a. The result can be seen in Fig. 3a and highlights that both expressions are completely equivalent in terms of functional shape. We should stress that in Fig. 3 no additional fitting has been done to make the two frameworks collapse. The harmony between these two expressions is even more remarkable if one considers that Eq. (2) was found on basis of a numerical 3D-VRH model, while (behavior that functionally resembles) Eq. (1) has been derived for various models of conduction, only one of them being related to 3D-VRH. Figure 3b shows the collapse of the same data set when the conductivity is plotted as a function of *T*_*eff*_, cf. Fig. 2b: the synthetic data created from the US expression Eq. (1) shows a power law scaling of the conductivity in *T*_*eff*_. Since conductivity and current are proportional and *T*_*eff*_ is, like the *x*-axis of Fig. 1b (*qV*/*k*_*B*_*T*), basically a measure for the combined effect of field and lattice temperature, the main plot in Fig. 3b contains essentially the same information as the plot of the scaled current in Fig. 1b (which is shown as an inset in Fig. 3b).

Following the above, it is in fact straightforward to show full mathematical equivalence of the limiting low- and high-field behavior of the Universal Scaling and Marianer-Shklovskii expressions. As discussed above, the limiting behavior of Eq. (1) is


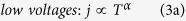



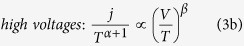


Transforming current density into conductivity using *j* ∝ *σV* and *β* = *α* + 1 yields


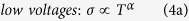



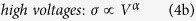


Inserting the Marianer-Shklovskii expression Eq. (2) into the experimentally found power law dependence of the conductivity on the effective temperature 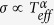 (cf. Figs 2b and 3b), we obtain


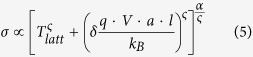


In the high- and low-field limits the first and second terms between the square brackets vanish, reducing Eq. (5) to Eqs (4b) and (4a), respectively. The equal exponents in Eqs (4a) and (4b) also explain why there is no longer a kink in the main panel of Fig. 3(b), whereas there is one in the inset where *j* instead of *σ* is plotted (c.f. Eq. 3).

Having established the experimental and functional connection between the US and MS, we can approach the question of the common physical background. To this end, theoretical investigations have been done using kinetic Monte Carlo simulations. Kinetic MC can be seen as a simulated real-world experiment under idealized and simplified conditions and with the ability to control every aspect of the virtual sample. In our case, we simulate Coulombically interacting particles performing nearest neighbor hopping (NNH) on a regular lattice with random site energies. Further details can be found in the methods section below and ref. 30. In view of previous experimental work on the same PEDOT:PSS materials, that indicate hopping/percolative transport in the in-/out-of-plane directions^31^,^32^, this is a logical choice. The use of NNH is justified in the present MC simulations in which we only consider high temperatures and high carrier concentrations. For an analytical treatment of NNH without Coulomb interaction see ref. 33. A further consequence of the use of NNH is that our results are independent of localization length, which is not a relevant parameter to NNH - in our work *a* refers to the lattice parameter.

The premise of the simulations used in this work is to reduce the number of assumptions and parameters to an absolute minimum, making the results as transparent as possible. The model will be shown to be sufficient to qualitatively and even quasi-quantitatively reproduce the experimentally obtained results over a wide range of parameters. For the simulations we used a commonly accepted value for the width of the Gaussian disorder of *σ* = 0.1 eV, a (relative) concentration *c* = 0.1, which roughly corresponds to a 1:2.5 PEDOT:PSS ratio and a PEDOT ionization fraction around 1/3, and a lattice constant *a* = 1.8 nm. Henceforth all simulation data presented in this work was calculated using these parameters unless stated otherwise. The specific morphological complexity of PEDOT:PSS is ignored, which not only facilitates computation but also warrants the relevance of the results to organic semiconductors in general. In order to keep calculation times within reasonable limits, only temperatures above room temperature are used.

**Figure 4:**
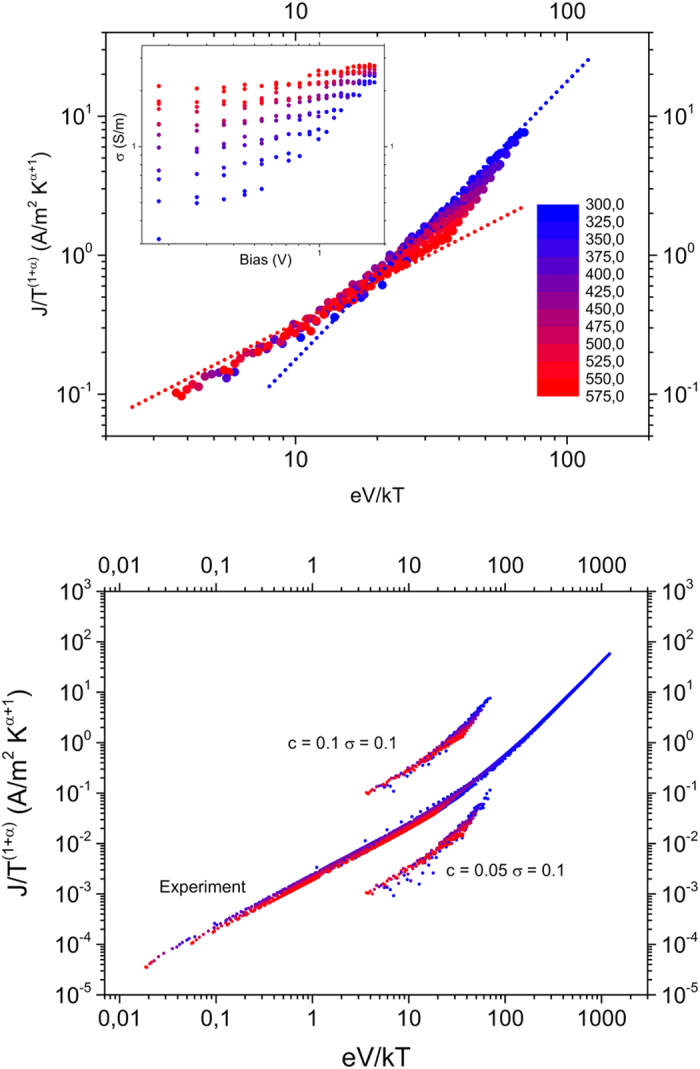
Universal scaling of simulation data. (**a**) Universal line of simulated current data scaled according to Eq. 1 with *α* = 2.0. The inset shows a plot of conductivity vs. bias voltage for various temperatures indicated by the color scale in the main panel. (**b**) Same as panel (**a**) with the addition of experimental data for PEDOT:PSS 1:2.5 and simulation data for two concentrations. The dotted lines are guides to the eye and represent an inclination of 1 (red) and 2 (blue) corresponding to the 2 regimes described above.

We performed the same ‘universal’ scaling procedure as before, e.g. from Fig. 1a,b, on the raw simulation data in the inset of Fig. 4a. The resulting curve is shown in the main panel of Fig. 4a. The spread in the collapsed curve appears to be large at first glance, but due to the fact that only a limited field and temperature range can be accessed in the simulations, a greatly magnified version of Fig. 1b is obtained. Where experimentally we could cover 7 orders of magnitude, we can only cover 3 orders in simulations, with a relative spread that is in fact comparable to the experimental spread in Fig. 1b. Importantly, apart from the highest lattice temperatures where we cannot reach sufficiently high fields, the individual traces show a clear and shifting transition point from Ohmic to non-Ohmic behavior from high to low temperatures (see inset) that collapses onto the transition region between the power law limits in the main panel of Fig. 4a. Simulation and experiment are plotted together in Fig. 4b, and show a quasi-quantitative correspondence that is surprising given the fact that the simulations have not been fitted to the experimental results; the parameters were simply chosen to be physically meaningful.

Although an exhaustive investigation of the parameter space is beyond the purpose of the present work, a limited set of calculations with different disorder types and widths is shown in the SI and suggest the results are rather robust in terms of shape and width of the disorder, provided a strongly energy dependent DOS is used. For decreasing concentrations the compatibility of the numerical results with US and MS scaling becomes significantly less. Note that in all cases concentrations beyond the Boltzmann limit were used, in line with the experimental systems discussed in the introduction.

**Figure 5:**
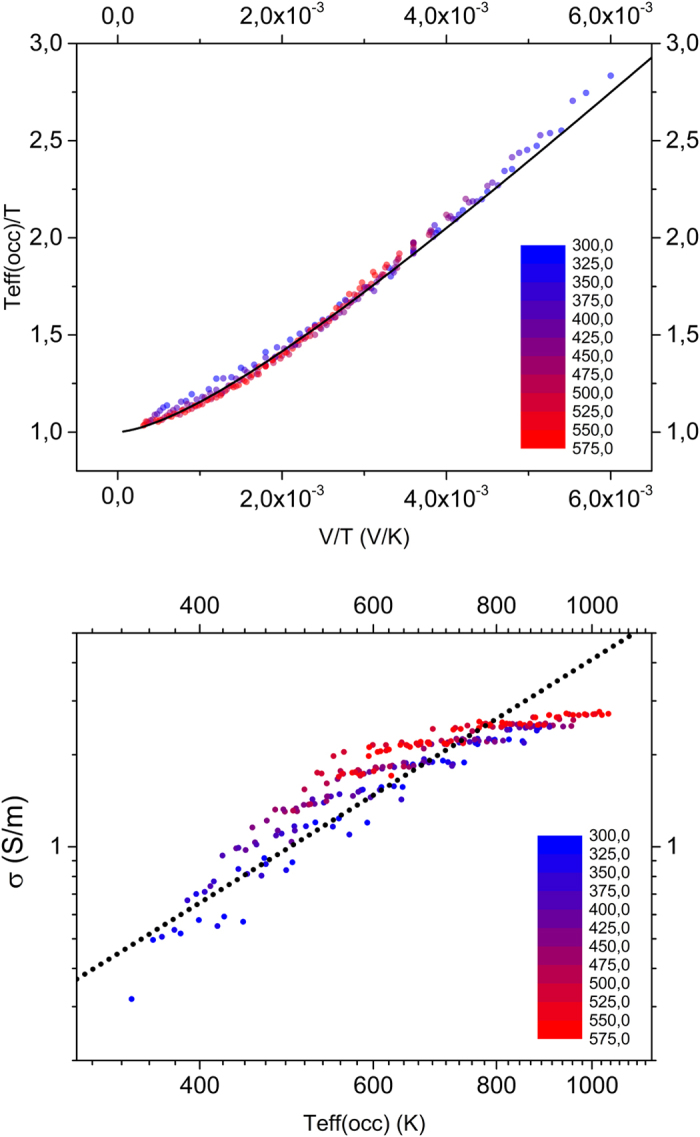
Effective temperature and correspondingly scaled conductivity of MC data. (**a**) Collapse of *T*_*eff*_ obtained from a fit of the Fermi-Dirac distribution to the energy-dependent occupation probability of the simulation data with a Gaussian disorder of 0.1 eV. The solid line represents a fit of the MS framework (Eq. 2) to the simulation data using *δ* = 0.67, *ς* = 1.5 and *a* = 0.9 *nm*. (**b**) Power law scaling of the conductivity with *T*_*eff*_. The dotted line corresponds to the power law relation 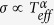 with *α* = 2.

From the MC output the occupation probability can be calculated as a function of site energy. Fitting the occupation probability to the Fermi-Dirac distribution function, 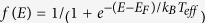, yields the effective temperature as a function of field and lattice temperature, *T*_*eff*_ (*F*, *T*). This method follows common practice and is equivalent to the way Marianer and Shklovskii obtained *T*_*eff*_ in their original work^19^,^21^,^22^. This method assures that, at least in the numerical simulations, *T*_*eff*_ is a proper measure of the characteristic energy of the charge carriers. In Fig. 5a *T*_*eff*_ is plotted against the field, scaled in the same manner as in Figs 2a and 3a and fitted with the MS expression Eq. 2. Excellent agreement between the MS framework (solid line) and the simulation data (filled circles) is observed. In Fig. 5b, the conductivity is plotted against *T*_*eff*_. Unlike for the experimental and synthetic data in Figs 2b and 3b, where the effective temperature was obtained from the Ohmic mobilities, a collapse of the MC conductivity data to a single curve is not trivial for the used procedure of calculating *T*_*eff*_ and in fact shows a significant spread. Hence, only an approximate power law-relation between conductivity and effective temperature can be established, as indicated by the dotted line in Fig. 5b. This observation is in line with earlier works concluding that the effective temperature is an approximate concept only^23^,^24^,^25^. However, it will be shown below that this approximate power law behavior is sufficient for the purposes of this work. Interestingly, when *T*_*eff*_ is obtained from the mobility of the MC data, i.e. following the procedure used to analyze the experiments, the field- and temperature-dependence of *σ* does collapse to a single power-law curve similar to Figs 2b and 3b, as is shown in the SI.

Having established both the functional equivalence of the US and MS frameworks and the applicability of our MC calculations, we shall now address a possible common physical background leading to the phenomenology that is characteristic for US and MS. Based on the knowledge that *T*_*eff*_ represents a characteristic energy of the charge carriers and the fact that the concept of *T*_*eff*_ is based on the heating of the charge carrier distribution, we start from the heat balance for the charge carriers in presence of an external field


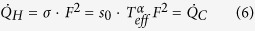


where the left hand side is the Joule heating per unit volume, which equals the energy loss to the lattice per unit time and volume 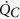. The second equality stems from substituting the conductivity with the experimentally obtained relation 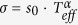 (cf. Figs 2b and 3b), where *s*_0_ is a proportionality constant.

The cooling or relaxation of the charge carrier distribution (at *T*_*eff*_) occurs via energy exchange with the lattice (at *T*_*latt*_). Since *T*_*eff*_ represents a characteristic energy of the charge carriers, the energy lost to the lattice per unit time and volume can be approximated by the time derivative of the Boltzmann energy of the charge distribution in absence of external heating,


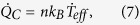


with *n* the number of charge carriers per unit volume. Equations (6) and (7) are based on the assumptions that the Ohmic conductivity has a power law dependence on temperature, and that *T*_*eff*_ represents the characteristic charge carrier energy and can be used to describe the conductivity of the system. In view of earlier work discussed above and our results presented so far, these assumptions seem reasonable.

When the system has reached steady-state the heating power is equal to the cooling power 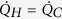 and we can write the heat balance


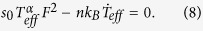


Solving this differential equation analytically requires an Ansatz for 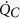 or, equivalently, a time-dependent expression of *T*_*eff*_.

**Figure 6:**
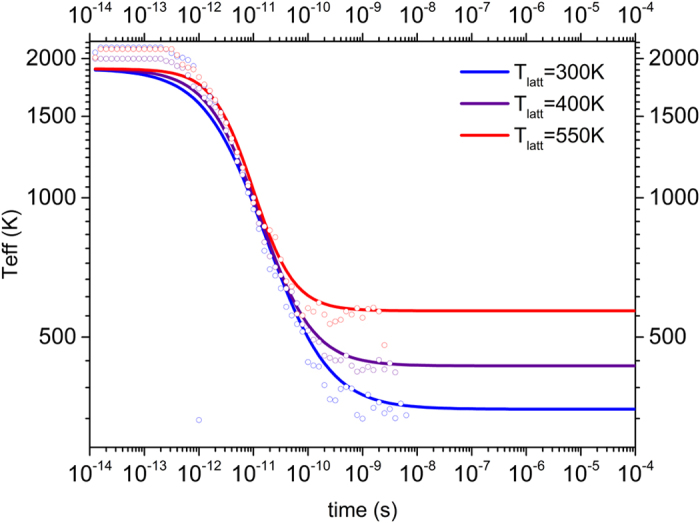
Temporal evolution of the effective temperature of the charge carrier distribution following a step in lattice temperature. At *t* = 0 the lattice temperature is stepped from *T*_0_ = 1900 K to the value indicated in the legend. Symbols show the relaxation of *T*_*eff*_ as calculated with MC. The full lines represent fits of Eqs (8) and (9) with a Gaussian disorder of 0.1 eV, *c* = 0.1, *α* = 2, *ϑ* ~ 1.0 ± 0.2 and *τ* ~ 10^−11^ *s*^−1^ to the simulation data.

We use our MC model to find an expression that adequately describes the relaxation of *T*_*eff*_. We define the same system as before and relax it to a high lattice temperature *T*_0_. At time *t* = 0 we step *T*_*latt*_ from *T*_0_ to a lower value and monitor the temporal evolution of the temperature of the charge carrier distribution *T*_*eff*_, plotted as open circles for different final temperatures in Fig. 6. By inspection of this data we obtained the following purely phenomenological expression, which obeys the condition *f*(0) = 0:


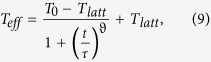


Here, *τ* is a relaxation time constant and *ϑ* is a stretching exponent; both are obtained from a fit of Eq. (9) to the simulation data, as shown in Fig. 6. The result is plotted as full lines in Fig. 6 for different final lattice temperatures, showing a reasonable approximation to the simulation data, especially in the relevant temperature range below ∼1000 K.

It is noteworthy that at low concentrations, i.e. in or close to the Boltzmann limit, the relaxation of *T*_*eff*_ deviates from Fig. 6, as shown in SI Figure 17. Instead, a double power law decay curve is found which cannot be fitted with our empirical expression Eq. (9). The fact that our empirical model breaks down at low concentrations is consistent with the previously discussed incapability of our numerical simulations to reproduce US and MS at lower concentrations. Additionally, when Coulomb interaction is not considered we find that Eq. (9) can only reproduce the relaxation of *T*_*eff*_ with *ϑ* > 1, also leading to a failure of the empirical model to consistently reproduce US and MS. The details of the relaxation process under different circumstances as well as the connection to US and MS are topic of ongoing work.

**Figure 7:**
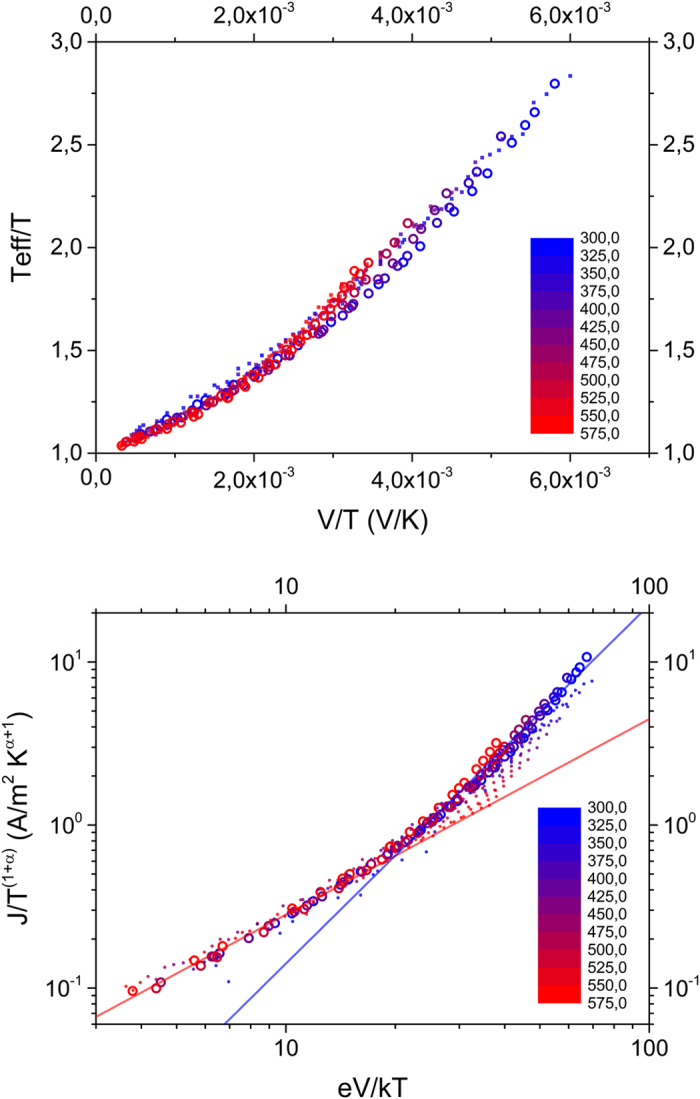
MS and US behavior of the heat balance model for current heating. (**a**) Effective temperature vs. field scaled with temperature. The dots represent the same MC data as Fig. 5a, the open circles are calculated from the heat balance model. (**b**) Current data from MC simulation (dots) and heat balance model (open circles) rescaled according to Eq. (1).

The energetic relaxation of charge carriers in amorphous systems in the Boltzmann limit has been studied before for the case of exponential DOS^34^ and Gaussian DOS^35^. Our results indicate that the relaxation time increases with decreasing temperature in agreement with ref. 35. Remarkably the relaxation time constant from the fit to the MC results has roughly the same value as the attempt frequency used in the MC simulation. Inserting Eq. (9) into the heat balance Eq. (8) gives an expression that can analytically be solved for *F* as a function of *T*_*eff*_; the expression however is too lengthy for display here and gives little insight. It is given in the SI. In order to evaluate the field- and lattice temperature-dependence and to compare the empirical model to the MC simulations, we inserted the values of *T*_*eff*_ and *T*_*latt*_ from our MC simulations into the expression for *F*, SI Eq. (1). This gives *F* for every *T*_*eff*_, *T_latt_* combination. The result is plotted in Fig. 7a. The conductivity and current are then determined from 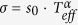 and *J* = *σ* · *F*, respectively, using the same values for *T*_*eff*_ that we entered into the expression for *F*. Independence of the results on *T*_0_, which has no physical meaning beyond being the starting temperature of the relaxation process, was assured, provided it was set to a reasonable value, in our case 1900 K. All other parameters in the heat balance model are determined by the simulations.

Equivalent to the way the experimental, synthetic and simulation data have been analyzed in Figs 1, 2, 3, 4, 5, *T*_*eff*_ and the scaled current from the heat balance model are plotted as lines in Fig. 7a,b together with the simulation data from Fig. 5a,b. We attribute the minor spread in the heat balance model curves to the imperfect fit of Eq. (9) to the data in Fig. 6. Nevertheless, the characteristics of both the Marianer-Shklovskii and universal scaling behavior are well-reproduced, showing that both can be understood as resulting from a balance between Joule heating and an (effective temperature dependent) relaxation. The crucial ingredient, leading to functional shapes resembling Eqs (1) and (2) is the algebraic time- or, equivalently, temperature-dependent relaxation rate shown in Fig. 6 and its approximation Eq. (9).

## Conclusions

In this work we have investigated two ‘universal’ scaling phenomena for the field- and temperature dependent conductivity of highly disordered organic semiconductors at high charge carrier concentration, the so-called universal scaling (US) and the Marianer-Shklovskii (MS) or effective temperature scaling. We have shown experimentally, numerically and analytically that phenomenologically the two scaling phenomena in fact describe the same functional dependence in temperature and voltage, with identical limiting behaviors. Experimentally US and MS scaling were observed in the out-of-plane transport in PEDOT:PSS thin films of various compositions. The observed behavior was quasi-quantitatively reproduced using a numerical nearest-neighbor hopping model with Coulomb interaction, high charge carrier concentration and energetic disorder as only ingredients. Finally, we derived an empirical model that shows that both scaling phenomena can have their physical origin in a simple heat balance of Joule heating and energy-dependent relaxation, under the condition that the Ohmic conductivity is a power law function of the (effective) temperature.

Although the described numerical and empirical models reproduce the main characteristics of US and MS scaling, they do not formally lead to the analytical expressions Eqs (1) and (2) that are commonly associated with these scaling behaviors. In fact, depending on the used input parameters, both the numerical and the empirical model show smaller or larger deviations from the ideal scaling behavior – something that is quite reminiscent of experimental reality in which many investigated systems show deviations of similar or even larger magnitude.

## Methods

### Experimental

PEDOT:PSS (Orgacon ICP-1050) with a PEDOT to PSS weight ratio of 1:2.5 was obtained from AGFA-Gevaert N.V. PEDOT:PSS weight ratios 1:6, 1:12, and 1:20 were prepared by adding PSS to the aqueous dispersions. Where necessary, water was added to obtain a solid content of 0.90 ± 0.04 w% and sonication was used to obtain homogeneous dispersions.

As substrates 4-inch silicon wafers with a 500 nm thermally grown silicon oxide were used. On this wafer, a 1 nm layer of chromium was thermally evaporated through a shadow mask, followed by 60 nm of gold. The root-mean-square (RMS) roughness of the bottom contact is about 0.7 nm over an area of 0.25 μm^2^. The two terminal junctions were photolithographically defined in an insulating matrix of photoresist, ma-N 1410 (Micro Resist Technology GmbH). After a pre-bake step to remove any remaining solvents, the layer was exposed to UV light with a Karl Süss MA1006 mask aligner to define the vertical interconnects, ‘vias’, with diameters of 5, 10, 20, 50, and 100 μm. After development the film was hard baked at 200 °C for at least 1 h. The wafer was subsequently cut in several pieces using a diamond tip pen. This allowed the processing of different PEDOT:PSS compositions on a single wafer, thereby eliminating lithographic variations that can affect device performance. A last step before layer deposition was cleaning of the bottom gold contacts with a PDC plasma cleaner (Harrick plasma) to remove any photoresist residuals. To obtain an equal layer thickness for all PEDOT:PSS ratios, the following spin coat parameters were used. The ramp-rate was 1000 RPM/s and the first spin coating step is 500 RPM for 5 s followed by 120 s of 2000 RPM (for 1:2.5), 1700 RPM (1:6), 1500 RPM (1:12), 1500 RPM (1:20). On planar test substrates these parameters led to layer thicknesses around 40 nm. After spin coating, the wafer was then immediately transferred to a vacuum oven to dry the film for at least 1 h. As top electrode, 100 nm of gold was evaporated through a shadow mask. This gold layer, apart from providing electrical contact with the measurement probes, also serves as a self-aligned mask for the removal of redundant PEDOT:PSS by reactive ion etching (O_2_ plasma). This step eliminates any parasitic currents from top to bottom electrode.

Measurements were performed in a high-vacuum probe-station (Janis research) at controlled temperatures between 5 and 300 K. Two types of *J-V* curves have been determined using a Keithley 2636a source-measure unit. Measurements often show transient changes, usually as irreversible steps in the conductivity with temperature. The measurements used for analysis were selected to contain no such changes.

### Numerical model

The MC experiments were performed in cube of variable side length *L* and periodic boundary conditions in all three dimensions. The localized sites are spatially distributed on a simple cubic lattice with an inter-site distance *a* = 1.8 nm and energetically with a Gaussian, exponential or constant distribution and varying degrees of disorder. The system contains *n* holes in a concentration *c* = *n*/*N* (*N* = *L*^3^/*a*^3^) and has no contacts, resulting in a constant carrier concentration during the course of one simulation. The size of the simulated system was chosen according to the relative concentration, i.e. small box size for high concentrations and large box size for small concentrations. The model example presented in this work with *c* = 0.1 was simulated in a 10 × 10 × 10 box, enclosing 100 charges; for *c* = 0.01 and *c* = 0.001 15 × 15 × 15 and 32 × 32 × 32 box sizes were used, enclosing 34 and 33 charges, respectively. For each set of parameters the data was averaged over 20 configurations. Coulomb interaction between all carriers was included using a commonly accepted value for the relative permittivity of 3.6. Also the Coulomb interactions of each carrier with the ‘twins’ of all other carriers that result from the 3D periodic boundary conditions have been included exactly up to a distance where the effect of the interaction becomes undiscernible – typically a cut-off of 5 box sizes was used.

We describe the charge transport by nearest-neighbor hopping of holes from an initial site *i* with energy *E*_*i*_ to a final site *j* with energy *E*_*j*_ with the hopping rate ν_*ij*_ according to the Miller-Abrahams expression


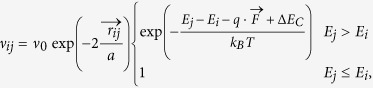


where ν_0_ is the attempt frequency, *r*_*ij*_ the vector connecting initial and final sites. The change in Coulomb energy is represented by Δ*E*_*C*_ and calculated by evaluation of the interaction of the moving charge with all other charges in the sample. The waiting time between hops and the direction of a hop are selected randomly according to the MC mechanism, using the rates of all possible transitions as weight factors. It should be mentioned that the validity of the MC simulations is restricted foremost by the field term in the Miller-Abrahams expressions. Too low fields result in currents that are in the order of the achievable statistical accuracy, while too high fields lead to a saturation of the mobility and a subsequent decrease. The reason for the latter effect lies in the fact that at some field (depending on the concentration and lattice temperature) the characteristic final site sits energetically at or below the initial site, so no further current gain is achieved by further increasing the field. Hence, to rule out any misleading effects we limited our theoretical investigations to a field range between these two limits.

## Additional Information

**How to cite this article**: Abdalla, H. *et al.* Effective Temperature and Universal Conductivity Scaling in Organic Semiconductors. *Sci. Rep.* **5**, 16870; doi: 10.1038/srep16870 (2015).

## Supplementary Material

Supplementary Information
