# Supplementary material for: Effective Temperature and Universal Conductivity Scaling in Organic Semiconductors
Source: Sci Rep. 2015 Nov 19;5:16870. doi: 10.1038/srep16870 (PMC4652272; doi:10.1038/srep16870)
Supplement: Supplementary Information [file srep16870-s1.pdf]

Supporting Information to

Effective Temperature and Universal Conductivity Scaling in Organic Semiconductors

Hassan Abdalla[1], Kevin van de Ruit[2] and Martijn Kemerink*[1,2]

[1] Complex Materials and Devices, Department of Physics, Chemistry and Biology, Linköping University, 58183 Linköping, Sweden.

[2] Eindhoven University of Technology, Department of Applied Physics, P.O. Box 513, NL-5600 MB Eindhoven, the Netherlands.

*corresponding author, martijn.kemerink@liu.se

Contents

Conductivity vs Bias and Universal Scaling for PEDOT:PSS 2

Effective temperature dependencies for PEDOT:PSS 3

Ohmic Conductivity vs. Temperature for PEDOT:PSS 4

Universal Scaling for simulation data with various DOS shapes 5

Effective temperatures from simulations for various DOS shapes 8

Simulated conductivity vs. effective temperature from mobility 12

Analytical solution to heat balance equations 13

Relaxation of $Teff$ without Coulomb interaction and in the Boltzmann limit 13

Conductivity vs Bias and Universal Scaling for PEDOT:PSS

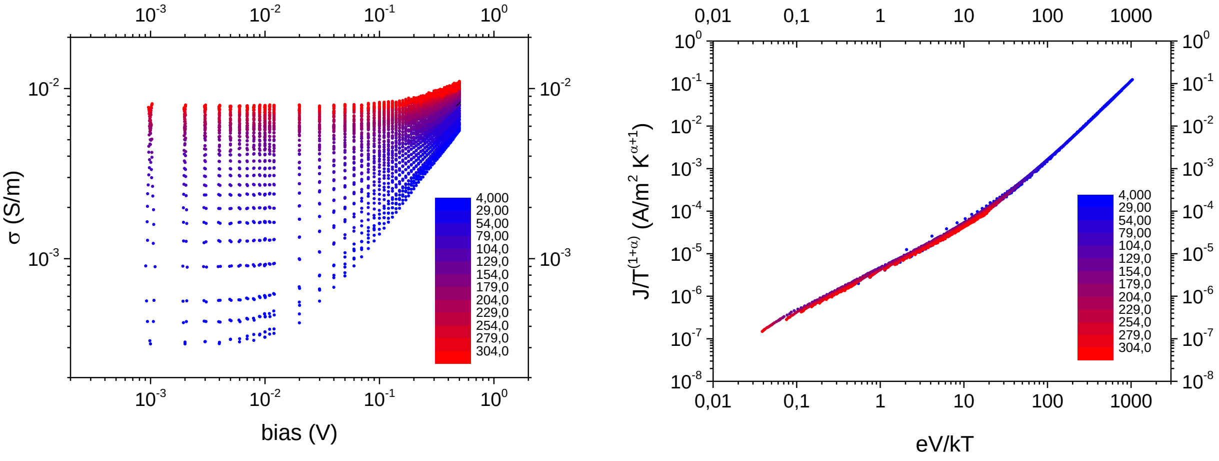

SI Figure 1 | PEDOT:PSS (w/w) ratio 1:6

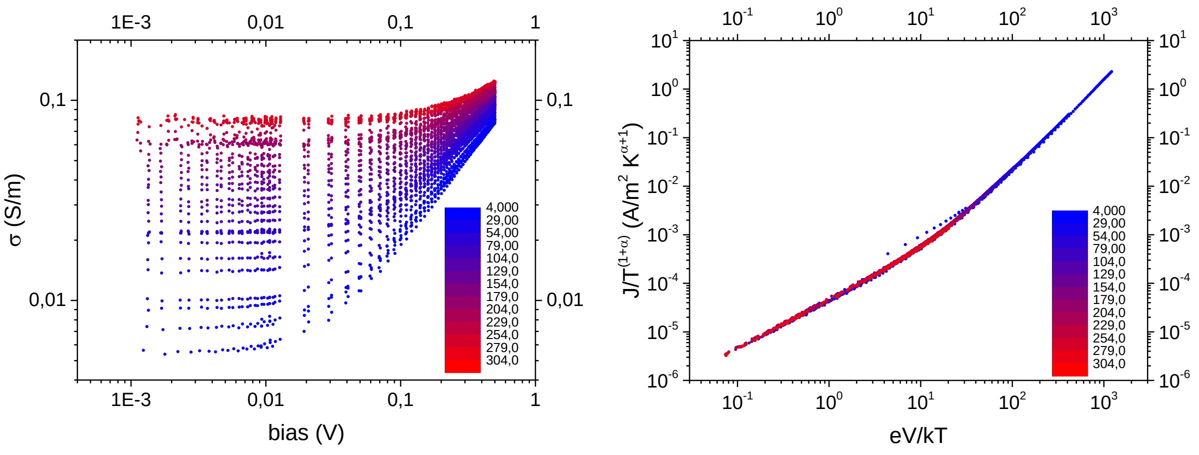

SI Figure 2 | PEDOT:PSS (w/w) ratio 1:12

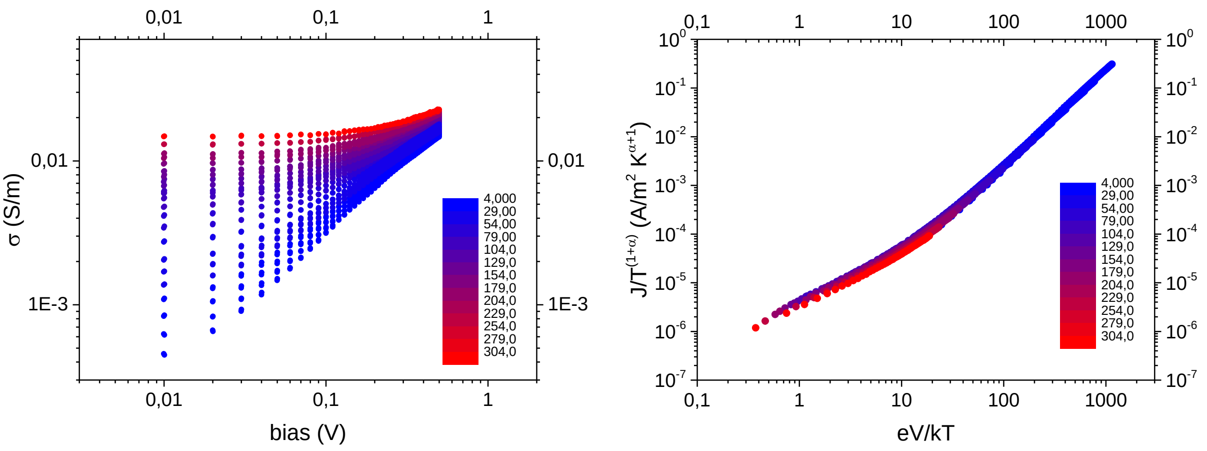

SI Figure 3 | PEDOT:PSS (w/w) ratio 1:20

Effective temperature dependencies for PEDOT:PSS

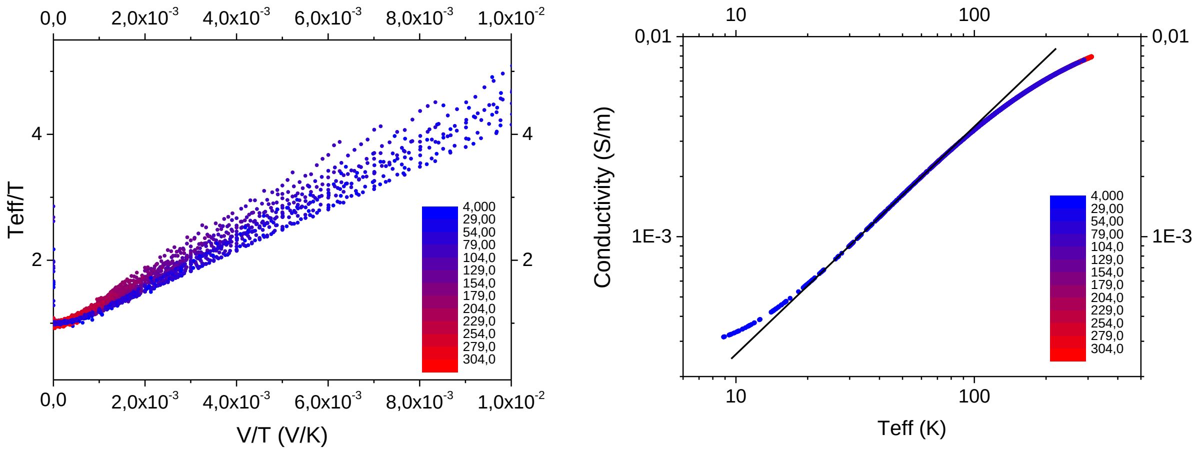

SI Figure 4 | Effective temperature dependencies for PEDOT:PSS (w/w) 1:6

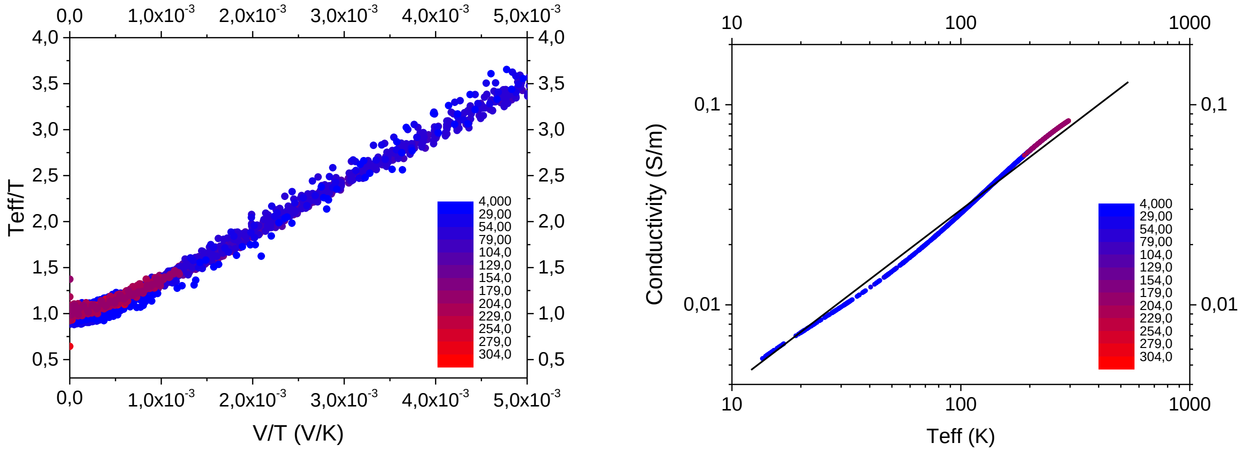

SI Figure 5 | Effective temperature dependencies for PEDOT:PSS (w/w) 1:12

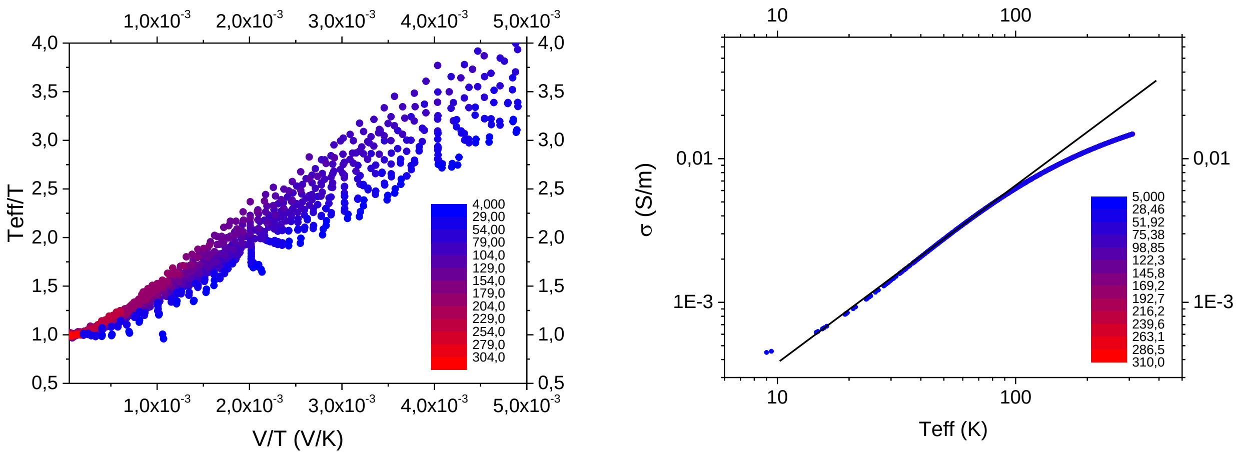

SI Figure 6 | Effective temperature dependencies for PEDOT:PSS (w/w) 1:20

Ohmic Conductivity vs. Temperature for PEDOT:PSS

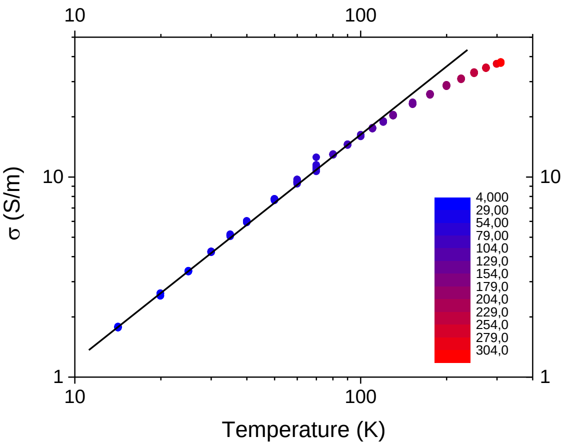

SI Figure 7 | Ohmic conductivity vs. temperature - measured at low bias voltages for PEDOT:PSS (w/w) 1:2.5

Universal Scaling for simulation data with various DOS shapes

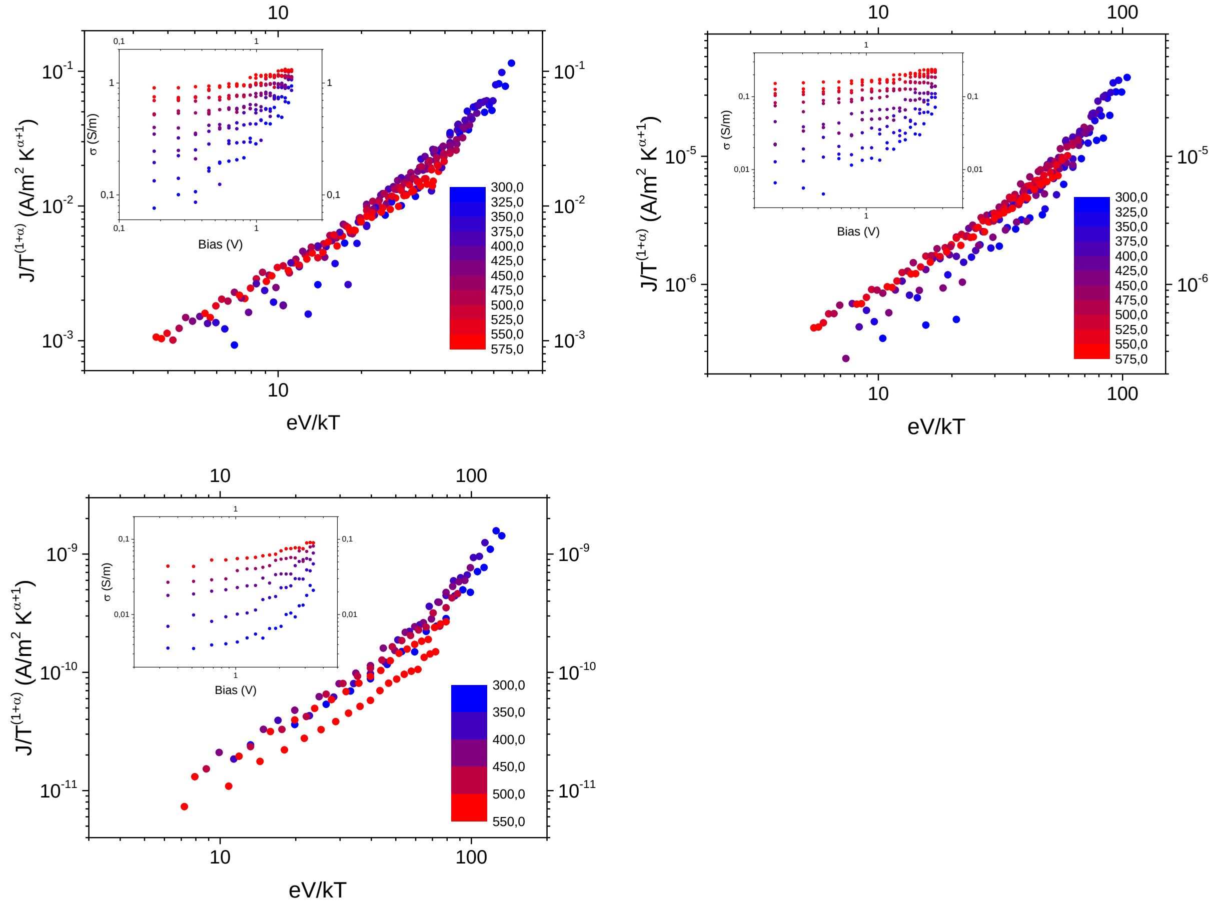

SI Figure 8 | Simulation data with Gaussian DOS of width 0.1eV. (a) Concentration = 0.05 **(b)** concentration = 0.01 **(c)** concentration = 0.005.

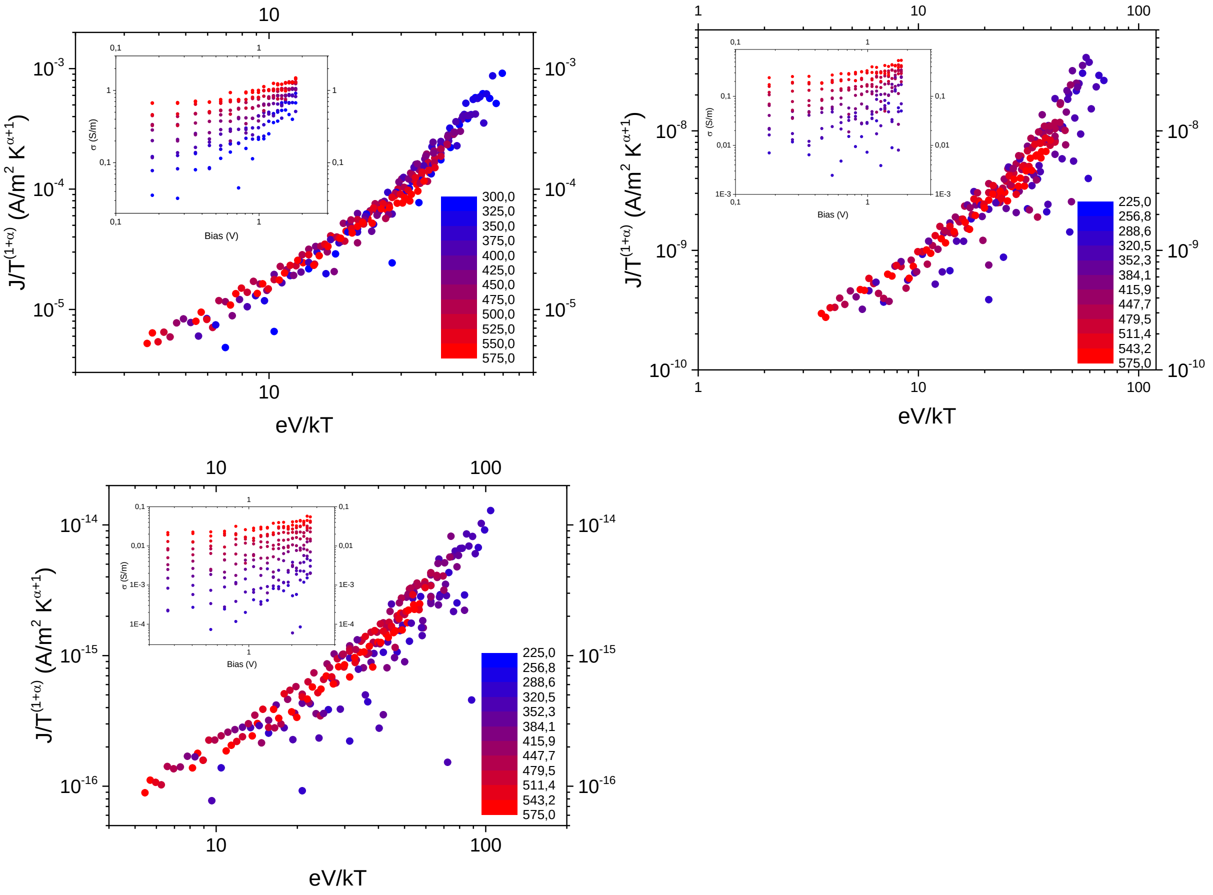

SI Figure 9 | Simulation data with Gaussian DOS of width 0.15eV. (a) Concentration = 0.1 **(b)** concentration = 0.05 **(c)** concentration = 0.01.

For all simulations with a Gaussian DOS, the shape Eq. (1), corresponding to universal scaling, seems to get less well reproduced with lower charge concentrations.

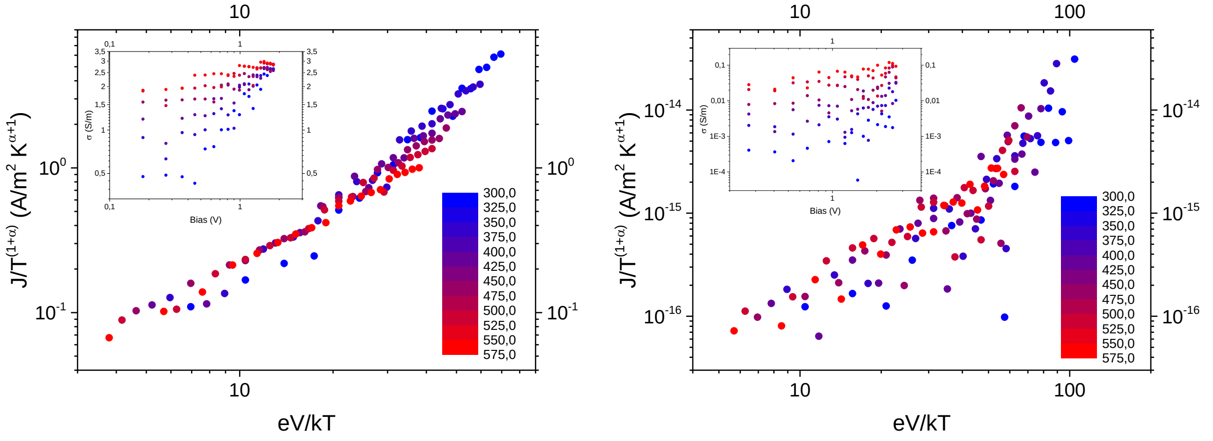

SI Figure 10 | Simulation data with exponential DOS of width 0.08eV. (a) Concentration = 0.1 **(b)** concentration = 0.01

Like for the simulations with a Gaussian DOS, the shape Eq. (1), corresponding to universal scaling, seems to get less well reproduced with lower charge concentrations.

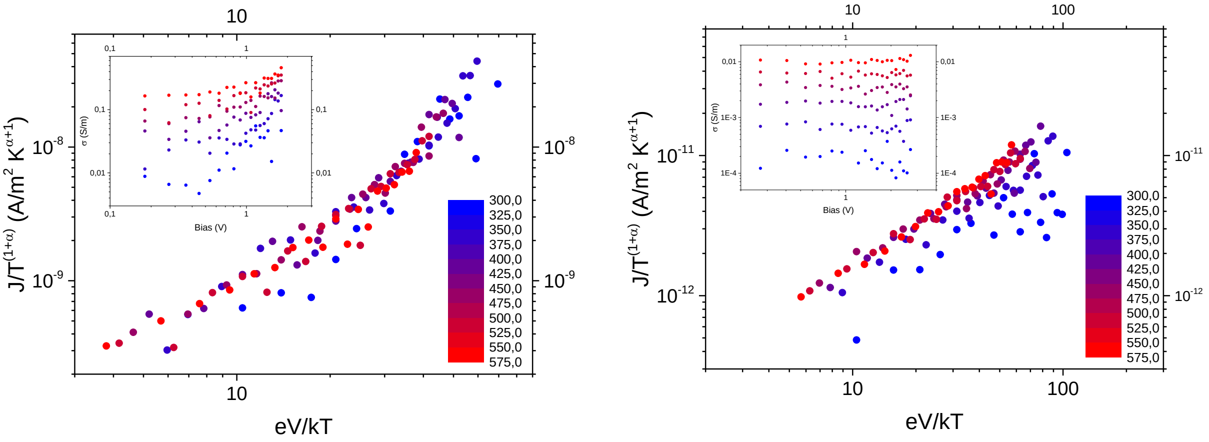

SI Figure 11 | Simulation data with a constant DOS of width 0.8eV. (a) Concentration = 0.1 **(b)** concentration = 0.01

For a constant DOS behavior resembling universal scaling is only visible for the highest simulated concentration (c = 0.1).

Effective temperatures from simulations for various DOS shapes

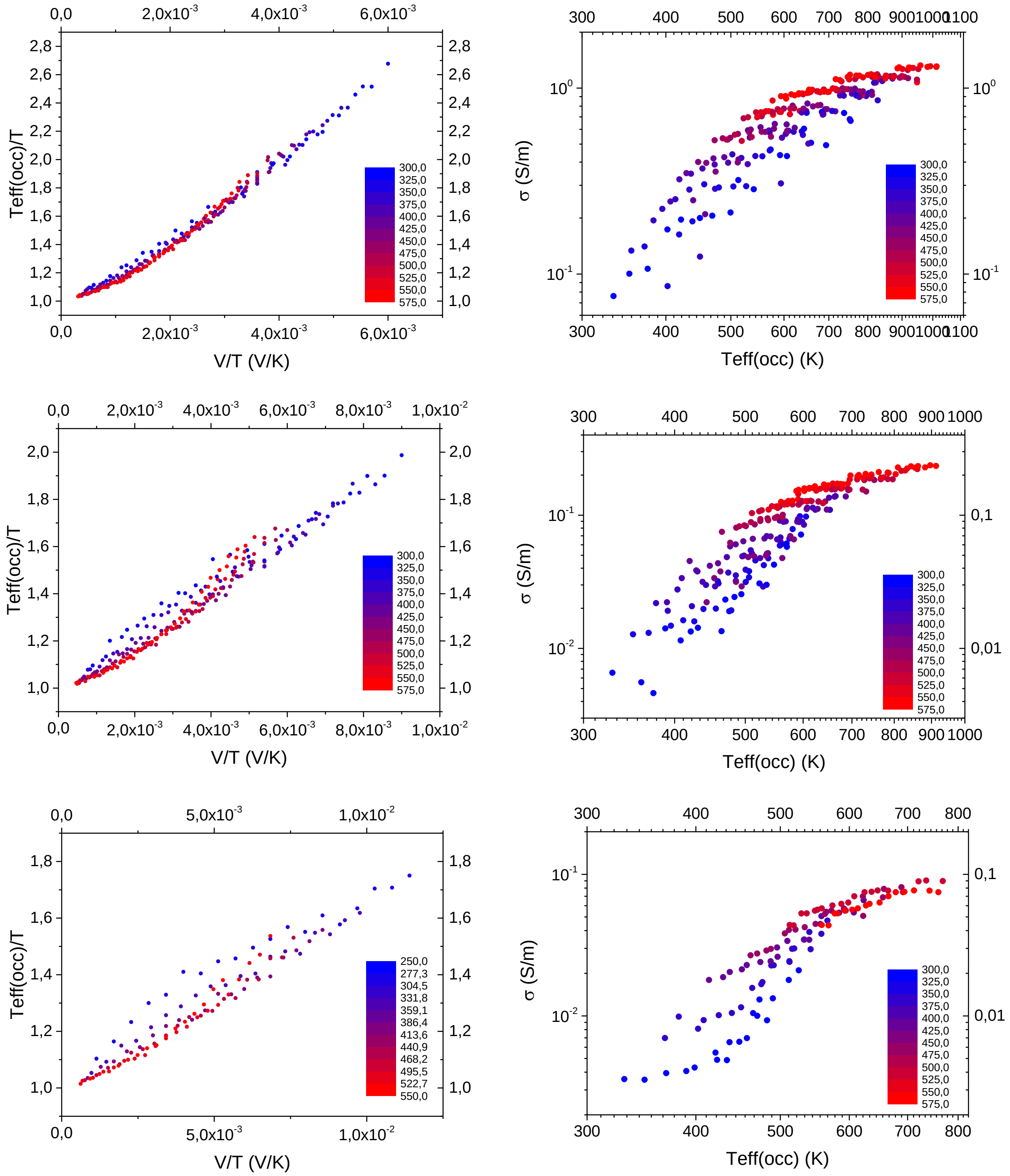

SI Figure 12 | Effective temperature dependencies from simulation data with Gaussian DOS of width 0.1eV. (a) concentration = 0.05 **(b)** concentration = 0.01 **(c)** concentration = 0.005

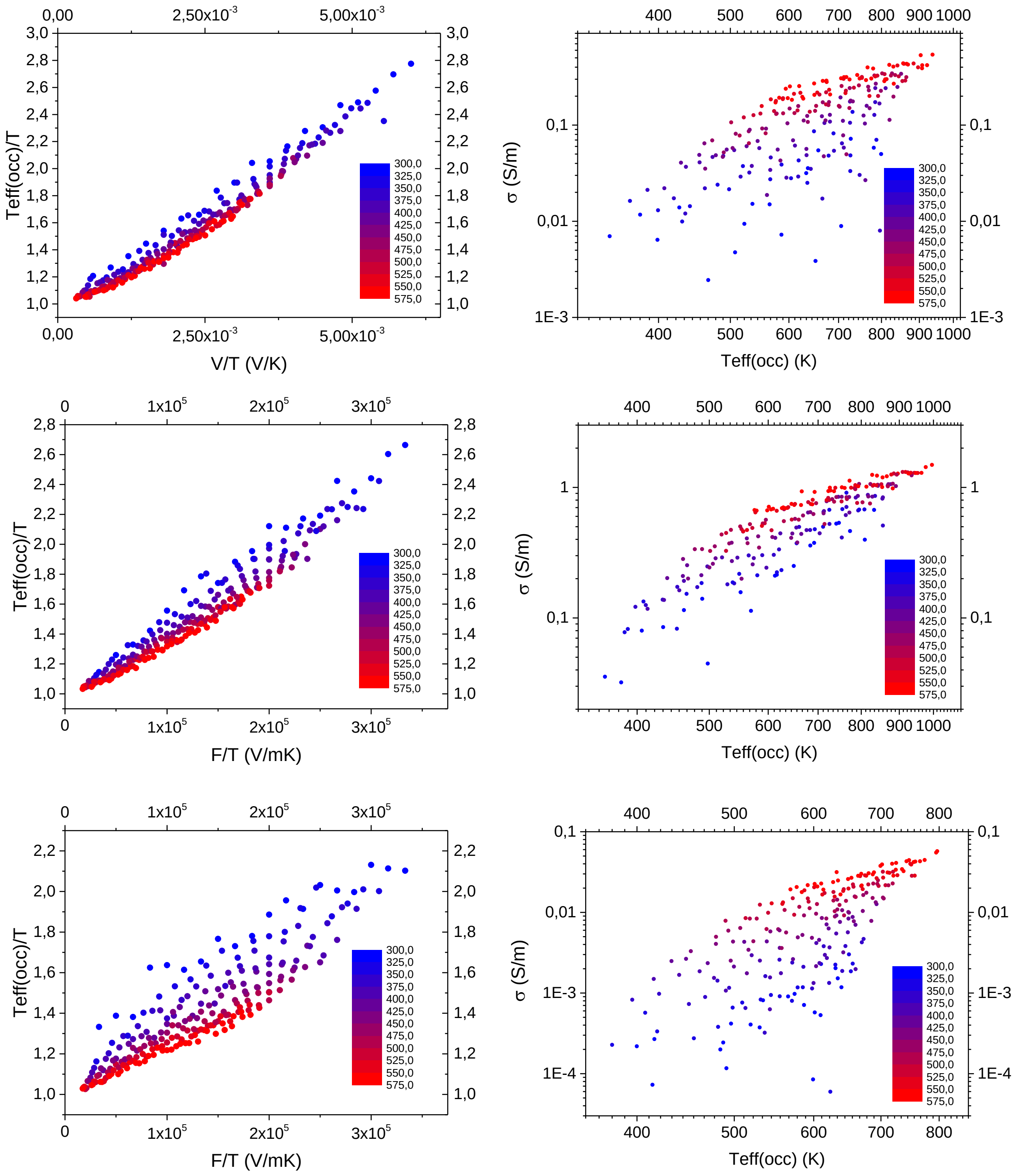

SI Figure 13 | Effective temperature dependencies from simulation data with Gaussian DOS of width 0.15eV. (a) Concentration = 0.1 **(b)** concentration = 0.05 **(c)** concentration = 0.01

For all simulations with a Gaussian DOS, both the shape Eq. (2) and the power law dependence $\sigma \propto T_{eff}^{\alpha}$ seem to get less well reproduced with lower charge concentrations.

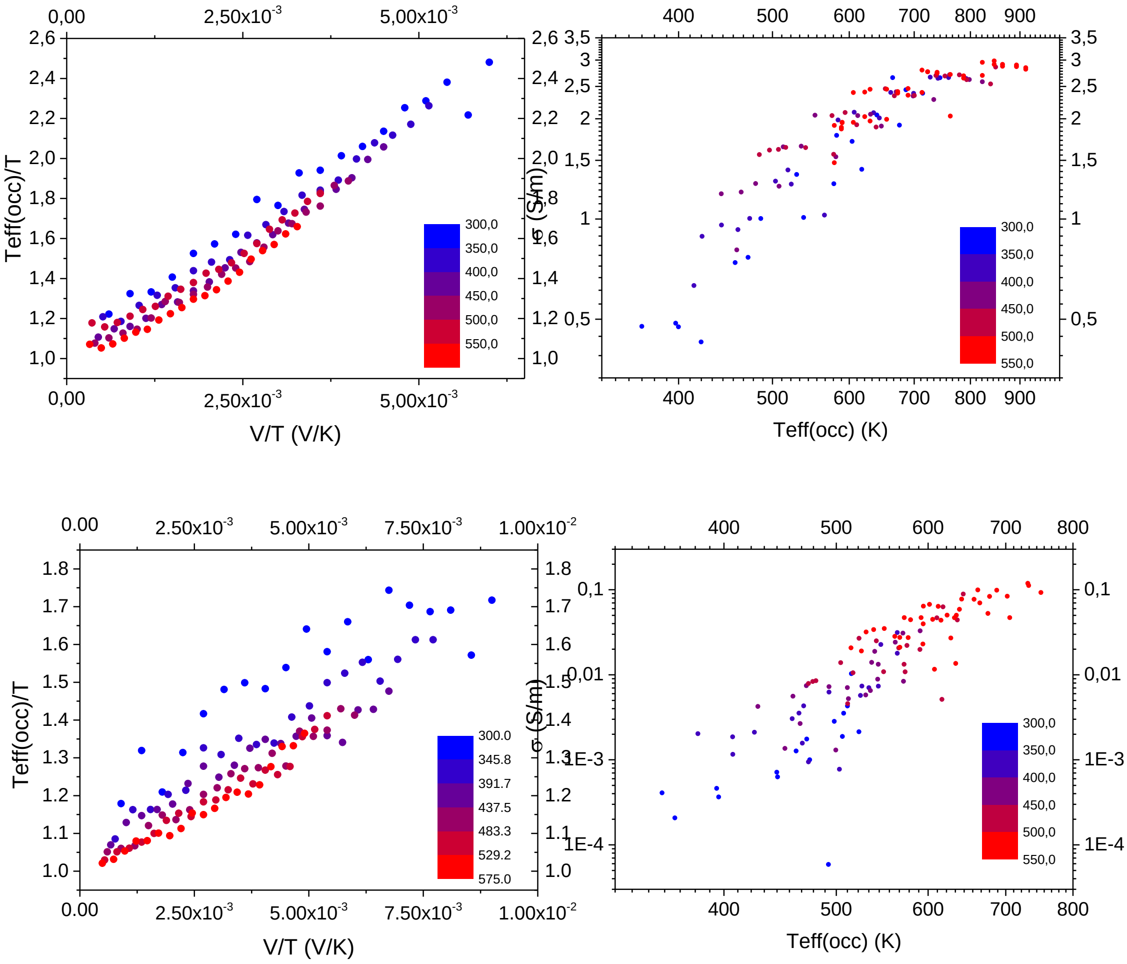

SI Figure 14 | Effective temperature dependencies from simulation data with exponential DOS of width 0.08eV. (a) Concentration = 0.1 **(b)** concentration = 0.05

Like for the Gaussian DOS, both the shape Eq. (2) and the power law dependence $\sigma \propto T_{eff}^{\alpha}$ seem to get less well reproduced with lower charge concentration.

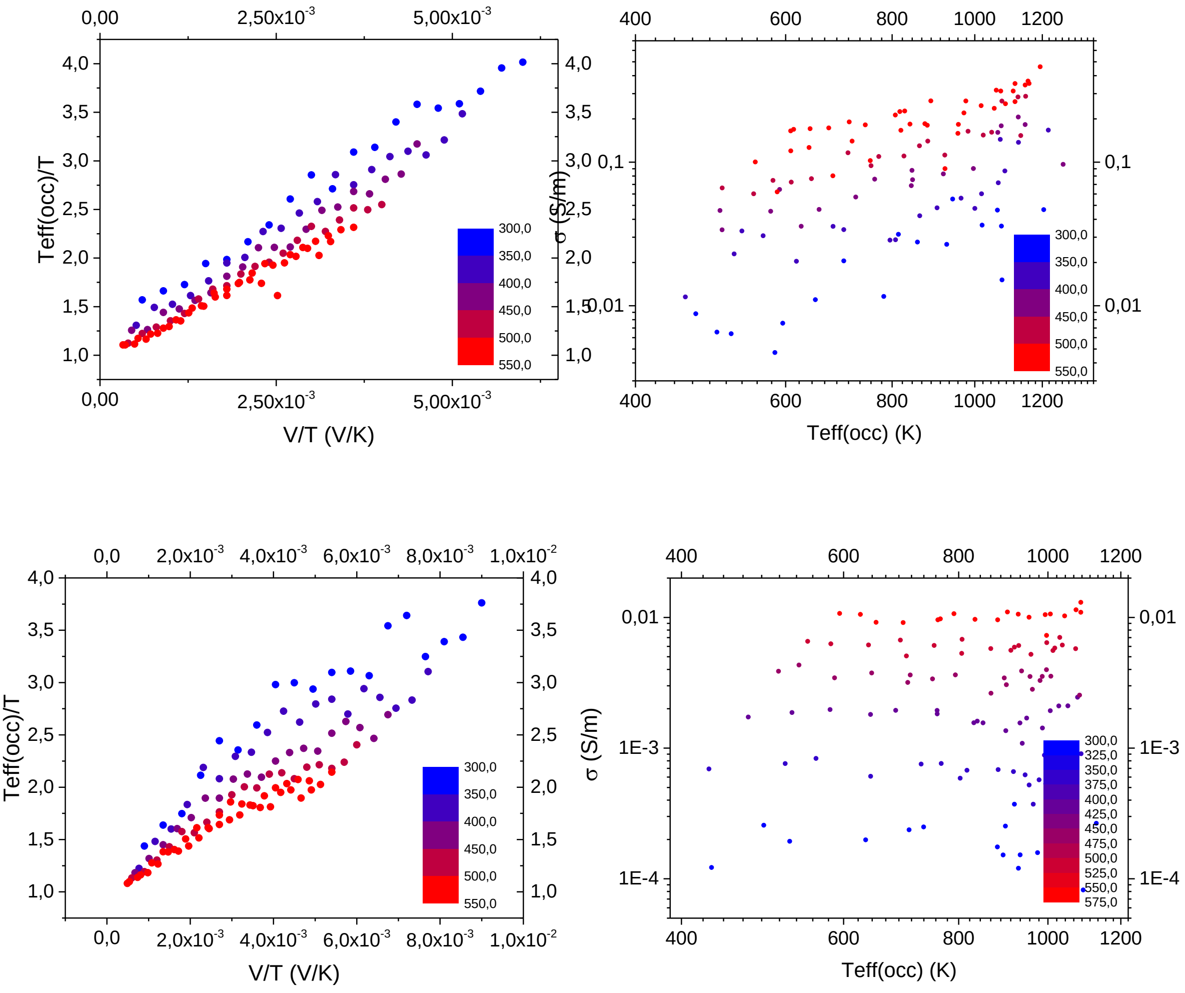

SI Figure 15 | Effective temperature dependencies from simulation data with a constant DOS of width 0.8eV. (a) Concentration = 0.1 **(b)** concentration = 0.05 **(c)** concentration = 0.01.

For a constant DOS MS-type scaling does not seem to work.

Simulated conductivity vs. effective temperature from mobility

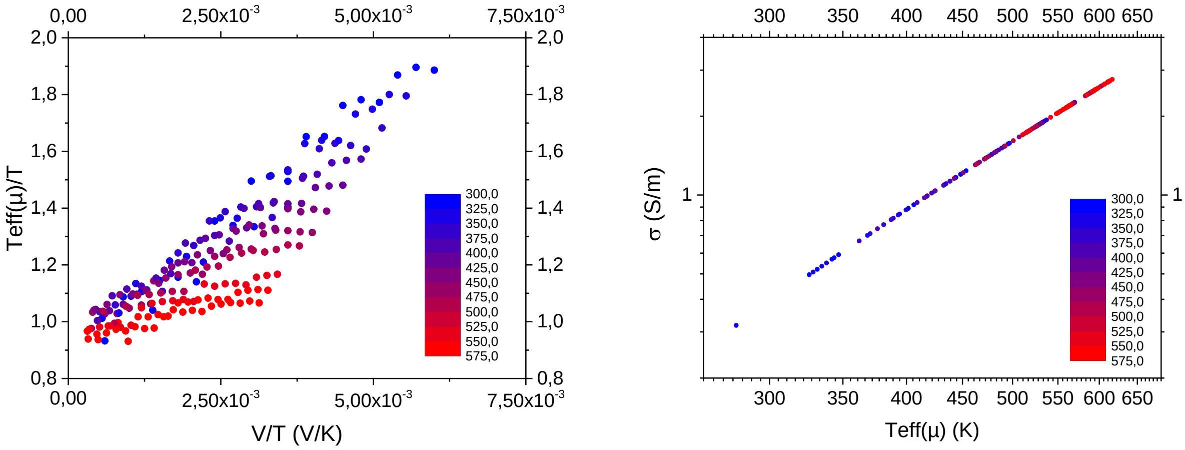

SI Figure 16 | Effective temperature dependencies for T_{eff} obtained from the mobility µ from simulation data with Gaussian DOS of width 0.1eV and concentration c = 0.1.

When the procedure used for the MS-analysis of the experimentally determined conductivity is applied to the conductivity obtained from the kinetic Monte Carlo simulations, a perfect power law fit of σ vs T_{eff} is obtained.

Analytical solution to heat balance equations

Inserting Eq. (9) into the heat balance Eq. (8) gives

$$F = \left(\frac{nk_B\vartheta}{s_0\tau}\right)^{\frac{1}{2}} T_{eff}^{-\alpha/2} \left(\frac{T_0 - T_{eff}}{T_{eff} - T_{latt}}\right)^{-\frac{1}{2\vartheta}} \left((T_{eff} - T_{latt})\frac{T_0 - T_{eff}}{T_0 - T_{latt}}\right)^{\frac{1}{2}}$$

The seeming violation of dimensions is caused by the dimension of s_0 depending on α.

Relaxation of T_{eff} without Coulomb interaction and in the Boltzmann limit

In SI Fig. 17 we plot the relaxation of T_{eff} as calculated from our MC simulation for the case of a low concentration c = 0.001 (open circles) as well as at c = 0.1 with Coulomb interaction switched off (filled circles) at different temperatures.

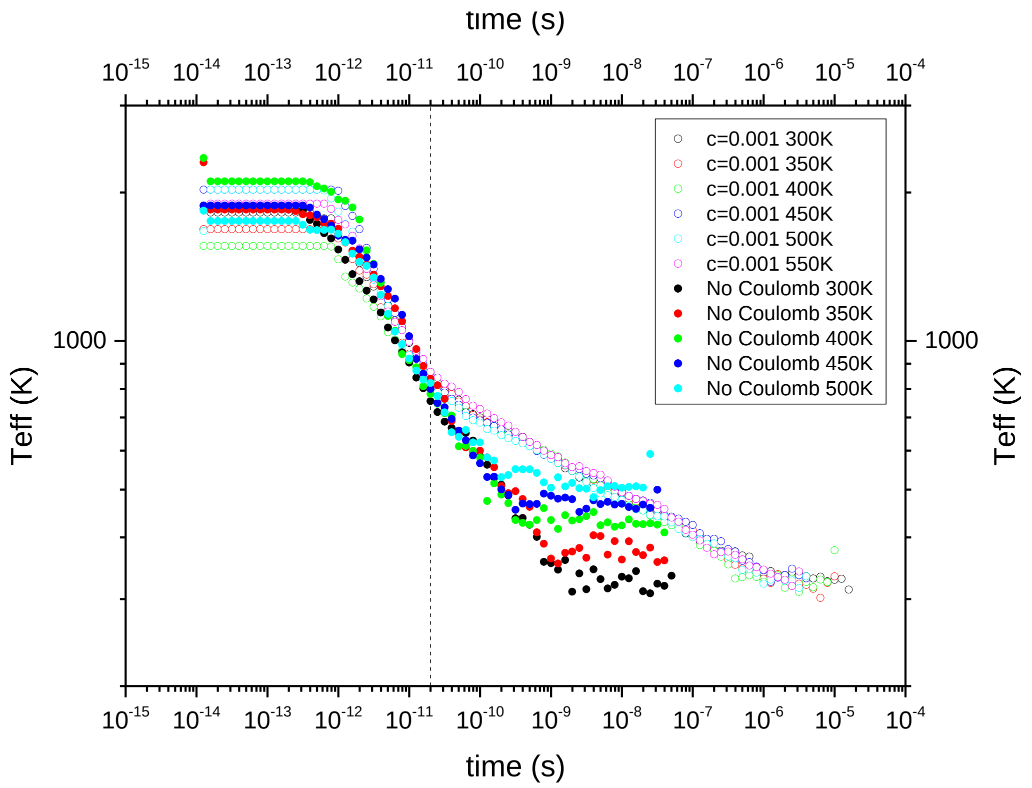

SI Figure 17 | Temporal evolution of the effective temperature of the charge carrier distribution following a step in lattice temperature for high concentration and no Coulomb interaction (filled circles) and low concentration including Coulomb interaction (open circles). A Gaussian DOS of width 0.1eV was used.

At low concentrations we observe a double power law decay. At early times before about 20 ps (vertical line in SI Fig. 17) the relaxation is dominated by energetically downward hops of the carriers, hence the independence on concentration, Coulomb interaction and lattice temperature. At the time indicated by the vertical line, the relaxation processes begin to distinguish. At high concentrations the steep decay continues uninterrupted to reaches steady state at the lattice temperature. We believe the reason for this to be the large number of carriers available with respect to the fraction of

deep tail states in the total density of states: the fact that relatively rare deep states are not yet occupied does not significantly show up in the ensemble average that therefore can appear to be 'quasi relaxed' at short times. In the case of low concentration charge carriers do need to find these rare deep tail sites, leading to an increasingly slow series of up- and downward hops as discussed in Ref. 34 of the main text.

Our ongoing research aims to gain more insight into this complex topic.
